# Supplementary material for: Rapid Purification and Formulation of Radiopharmaceuticals via Thin-Layer Chromatography
Source: Molecules. 2022 Nov 24;27(23):8178. doi: 10.3390/molecules27238178 (PMC9738419; doi:10.3390/molecules27238178)
Supplement: Supplementary file 1 [file molecules-27-08178-s001.zip › molecules-2020290-supplementary.pdf]

molecules

Supplementary Information

Rapid Purification and Formulation of Radiopharmaceuticals via Thin-Layer Chromatography

Travis S. Laferriere-Holloway [1,2], Alejandra Rios [2,3], Giuseppe Carlucci [1,3,4] and R. Michael van Dam [1,2,3,*]

[1] Department of Molecular & Medical Pharmacology, David Geffen School of Medicine, University of California, Los Angeles, CA, 90095, USA
[2] Crump Institute for Molecular Imaging, University of California, Los Angeles, CA, 90095, USA
[3] Physics and Biology in Medicine Interdepartmental Graduate Program, David Geffen School of Medicine, University of California, Los Angeles, CA, 90095, USA
[4] Ahmanson Translational Theranostics Division, Department of Molecular & Medical Pharmacology, University of California, Los Angeles, CA, 90095, USA
* Correspondence: mvandam@mednet.ucla.edu

Citation: Laferriere-Holloway, T.S.; Rios, A.; Carlucci, G.; van Dam, R.M. Rapid Purification and Formulation of Radiopharmaceuticals via Thin-Layer Chromatography. *Molecules* **2022**, *27*, 8178. https://doi.org/10.3390/molecules27238178

Academic Editor: Svend Borup Jensen

Received: 24 October 2022
Accepted: 18 November 2022
Published: 24 November 2022

Publisher's Note: MDPI stays neutral with regard to jurisdictional claims in published maps and institutional affiliations.

Copyright: © 2022 by the authors. Licensee MDPI, Basel, Switzerland. This article is an open access article distributed under the terms and conditions of the Creative Commons Attribution (CC BY) license (https://creativecommons.org/licenses/by/4.0/).

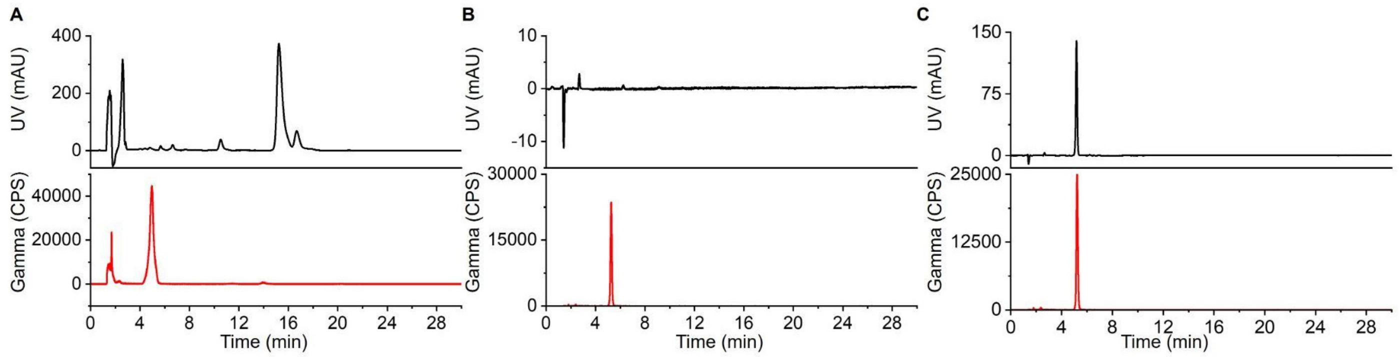

Figure S1. HPLC chromatograms of [18F]Fallypride samples. (A) Crude reaction product. (B) TLC-purified product. (C) Co-injection of TLC-purified product with Fallypride reference standard.

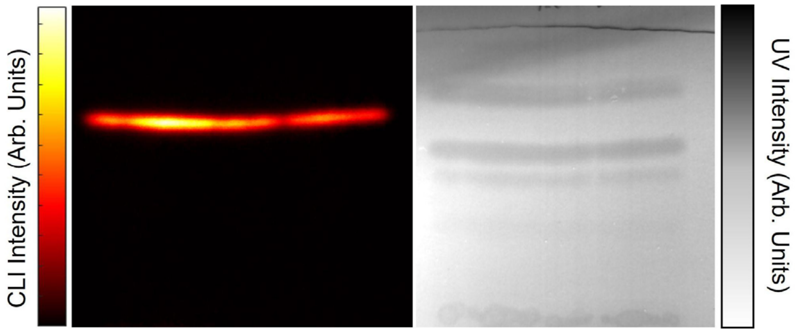

Figure S2. Images (Left: CLI; Right: UV) of crude [18F]Fallypride deposited on TLC plates using the streaking deposition method.

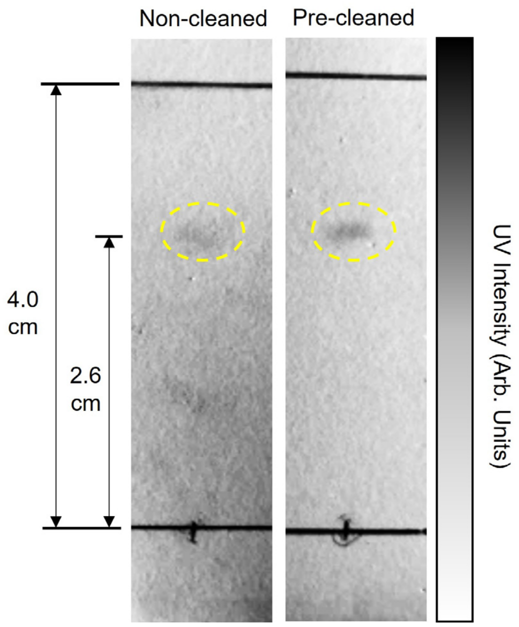

Figure S3. UV images of non-cleaned and pre-cleaned TLC plates after spotting with the PBR-06 reference standard and developing in the mobile phase for [18F]PBR-06. Yellow dashed circles denote the PBR-06 band. The non-cleaned plate appears to have additional regions with significant UV signals.

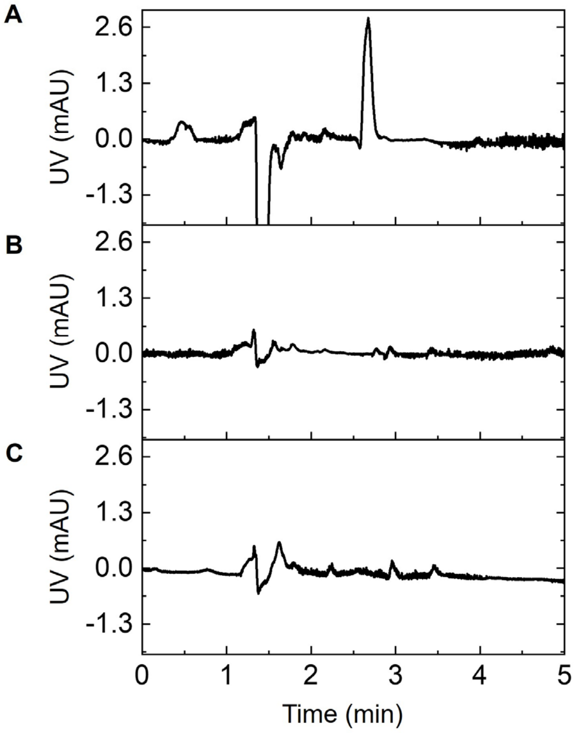

Figure S4. HPLC analysis (using the mobile phase for PBR-06) of mock samples obtained by silica collection and subsequent product extraction with saline from (A) non-cleaned and (B) pre-cleaned TLC plates. (C) Blank injection with fresh saline.

Section S1. Quality control testing methods

Quality control tests for final appearance (color and clarity), radionuclide purity and identity, bacterial endotoxins, filter integrity (via bubble point test), sterility, radiochemical and chemical purity were determined as previously described (cite). The remaining QC tests were performed as described below.

Molar Activity

Molar activity was estimated by quantifying the area under the curve (AUC) of the product peak in the HPLC chromatogram (UV signal) of the purified radiopharmaceutical. AUC was converted to a molar amount using a calibration curve generated using the reference standard (**Figures S5** and **S6**). Finally, the molar activity was computed by dividing the collected radioactivity of the product peak by the molar amount determined from the UV peak.

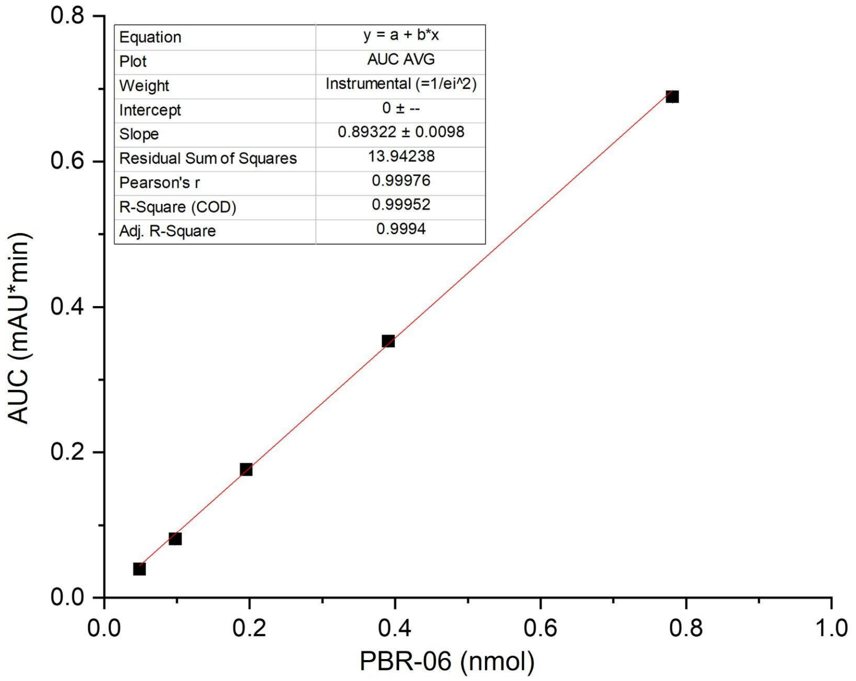

Figure S5. Calibration curve for PBR-06 (n = 3 for each data point).

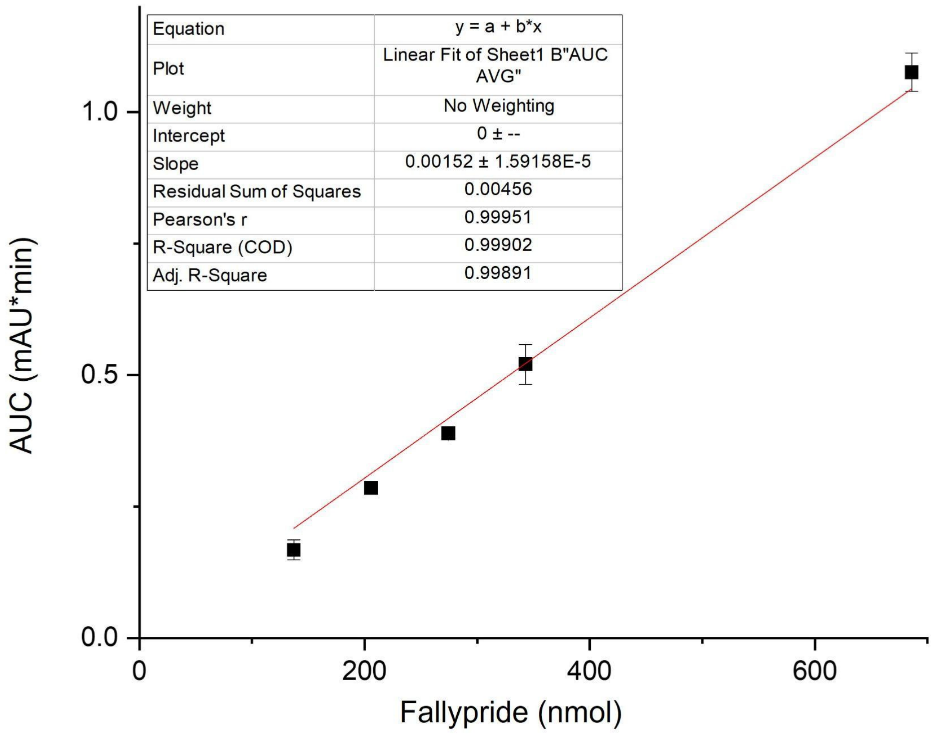

Figure S6. Calibration curve for Fallypride (n = 5 for each data point).

Residual $TBAHCO_3$

Residual TBAHCO3 has an acceptable limit of 104 mg/L for a 1 mL formulation volume. Two assays were performed to compare the level of TBAHCO3 in formulated radiopharmaceutical samples to a reference of 45 mg/L TBAHCO3.

Using the method described by Kuntzsch et al.[1], 2 µL of TBAHCO3 reference was spotted against formulated [18F]PBR-06 and [18F]Fallypride (2 µL) onto TLC plates. After drying under ambient conditions, 5 µL of 0.72 M NH_4OH (9:1 MeOH:H_2O (v/v)) was spotted on top of each sample. Dried samples were immersed in a sealed container to be subjected to staining with I_2 for a total of 2 min. Example images of these tests are shown for each radiopharmaceutical in **Figure S7**.

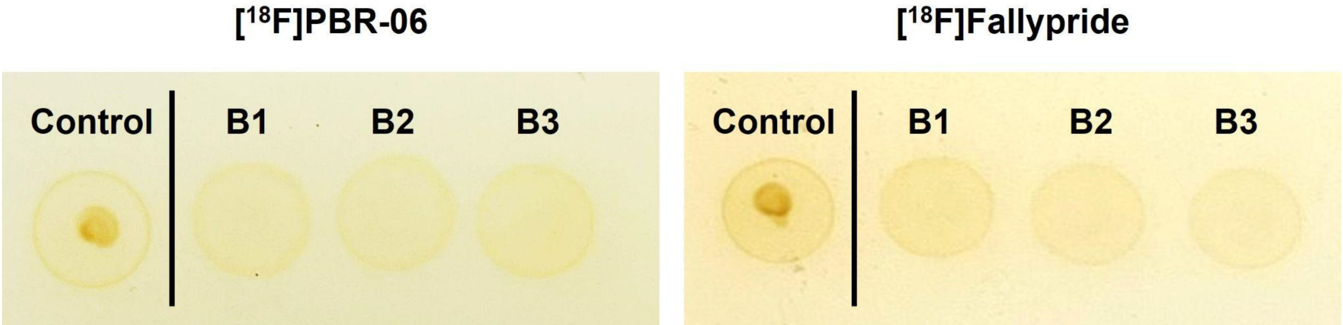

Figure S7. Images of iodine-stained plates to test for residual TBAHCO3. (Left) Reference (45 mg/L) compared with three batches (B1, B2, B3) of formulated [18F]PBR-06. (Right) Reference compared with three batches of formulated [18F]Fallypride.

Another test was performed using a recent method reported method using Dragendorf's reagent[2]. Similar to the method above, TBAHCO3 reference and radiopharmaceutical samples (2 µL) were spotted on TLC plates. Plates were dried, directly dipped into the stain, and removed immediately. Heating with a heat gun (setting 170 °C) revealed TBAHCO3 as a violet color, as shown in the example images in **Figure S8**.

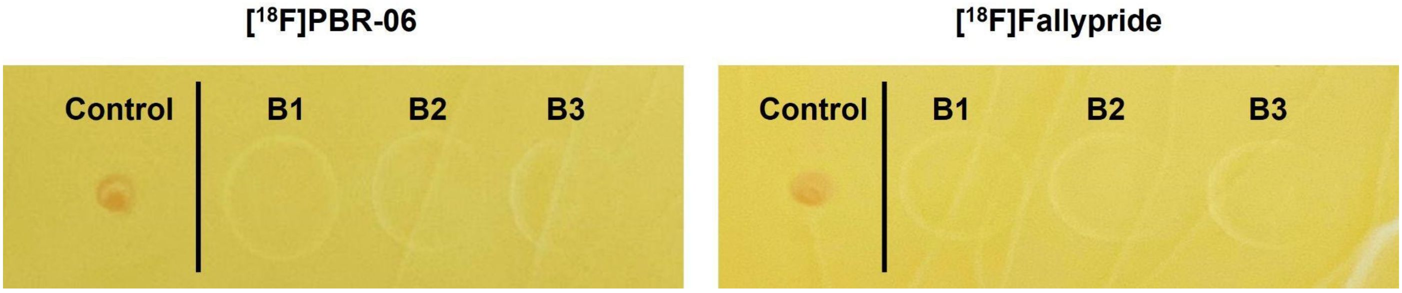

Figure S8. Images of Dragendorf-stained plates to test for residual TBAHCO3. (Left) Reference TBAHCO3 (45 mL/L) compared with three batches (B1, B2, B3) of [18F]PBR-06. (Right) Reference TBAHCO3 compared with three batches of [18F]Fallypride.

Residual solvent analysis

The concentration of residual solvents (MeCN, MeOH, Hexanes, $CHCl_3$, Et_2O, EtOAc, AcOH, and thexyl alcohol) was determined using gas chromatography with mass spectrometry detection (GC-MS).

GC-MS measurements were carried out using an Agilent 6890N GC, 5975 MSD, and 7683B autosampler. The instrument was controlled by Enhanced Chemstation software version E.01. The GC inlet was operated in split mode at 250 °C. UHP helium (Airgas West, Culver City, CA) was used as the carrier gas, with the flow rate set to 1.2 mL/min. GC separation was carried out on a 30m x 250 µm x 0.25 µm DB-Wax column (Agilent J&W). The GC oven was initially held at 40 °C, heated to 100 °C at 10 °C/min, and then heated to 230 °C at 30 °C/min. The MSD was operated in the SIM mode and used EI ionization.

Instrument response for known concentrations of all pure analytes in water was measured to determine the analyte concentrations in the unknown samples. A linear relationship was observed between the known concentration values and instrument response for these standard samples. The measured analyte response values for known and unknown samples were normalized to that of isopropanol used as the internal standard.

pH

The resultant formulation was assessed via commercial pH test strips (MColopHast, EMD Millipore, Darmstadt, Germany). Chemical indicators embedded into the tape change color in response to hydroxide and hydrogen ion concentrations. Samples images are shown in **Figure S9**.

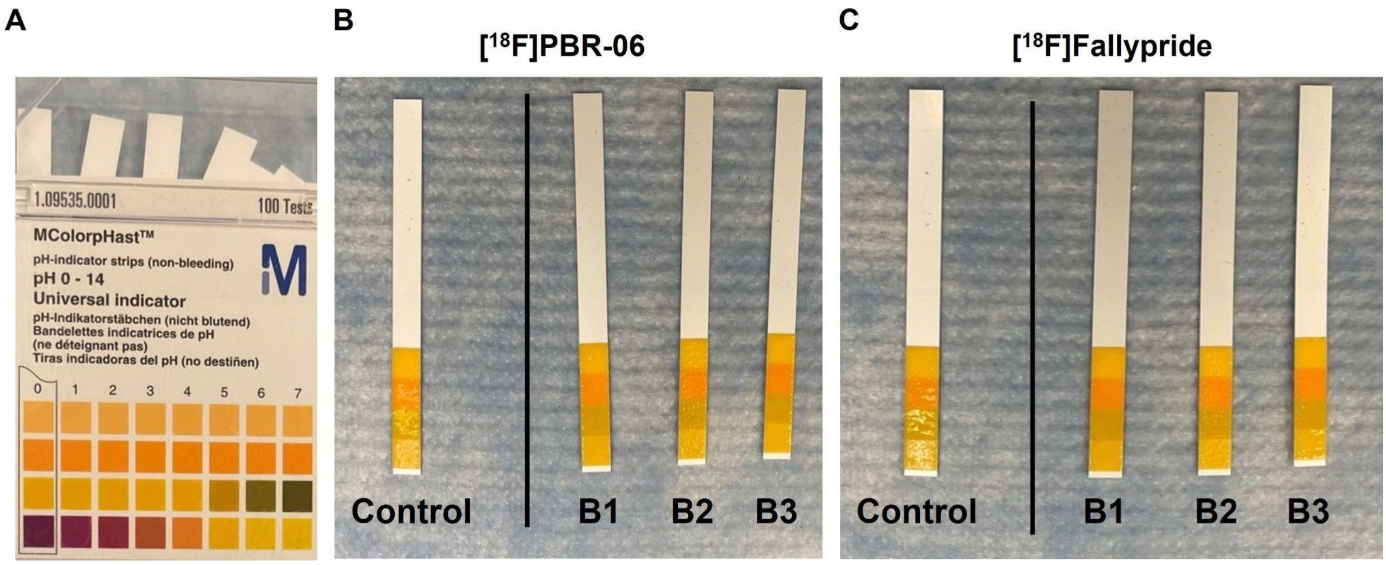

Figure S9. pH testing. (A) Color scale. (B) pH tests of saline (control) and three batches (B1, B2, B3) of TLC-purified [^{18}F]PBR-06. (C) pH tests of saline and three batches of [^{18}F]Fallypride.

Radionuclidic Identity

The identity of the radionuclide was determined by counting an aliquot of the formulated radiopharmaceutical in a calibrated dose calibrator over the course of 30 min. The following formula was compared to measured values to determine the radionuclidic identity:

$$T1/2 = -0.693 * (\Delta t)/ln(Af/A0)$$

where T1/2 = the half-life of the radionuclide, Δt = the change of time between two measurements, Af = final activity measured, and A0 = the initial activity measured.

Radiochemical Identity

Radiochemical identity was confirmed by co-injection of the purified radiopharmaceutical and corresponding reference standard and by comparing the retention times of the peaks in the UV and radiation signals.

Shelf-life

Radiochemical and chemical purity was assessed for batches via radio-HPLC after 120 min. A sample chromatogram for a sample of [^{18}F]PBR-06 is shown in **Figure S10**.

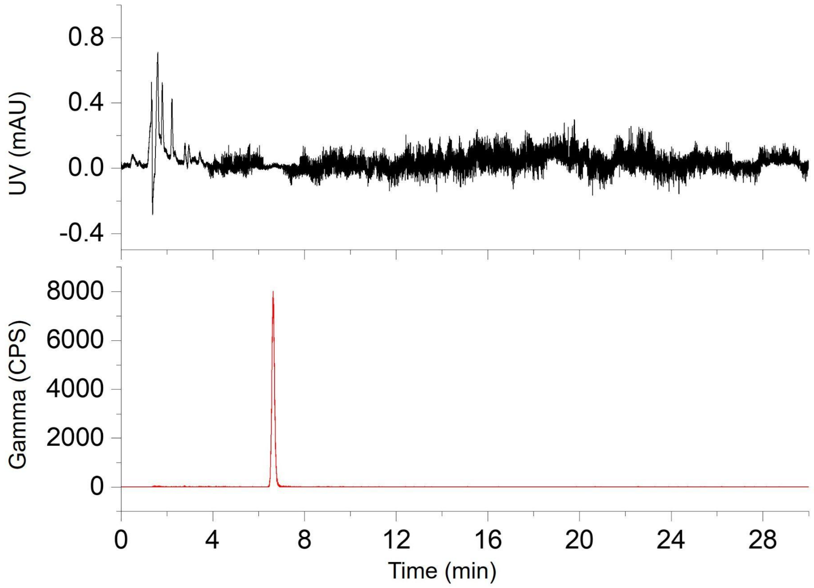

Figure S10. HPLC chromatogram of formulated [^{18}F]PBR-06 injected 120 min after the end of synthesis.

References

1. Kuntzsch, M.; Lamparter, D.; Bruggener, N.; Muller, M.; Kienzle, G.J.; Reischl, G. Development and Successful

Validation of Simple and Fast TLC Spot Tests for Determination of Kryptofix® 2.2.2 and Tetrabutylammonium in 18F-Labeled Radiopharmaceuticals. *Pharmaceuticals* **2014**, *7*, 621–633, doi:10.3390/ph7050621.

2. Tanzey, S.S.; Mossine, A.V.; Sowa, A.R.; Torres, J.; Brooks, A.F.; Sanford, M.S.; Scott, P.J.H. A Spot Test for Determination of Residual TBA Levels in 18F-Radiotracers for Human Use Using Dragendorff Reagent. *Anal. Methods* **2020**, *12*, 5004–5009, doi:10.1039/D0AY01565B.
